# Statistical Analysis Reveals Co-Expression Patterns of Many Pairs of Genes in Yeast Are Jointly Regulated by Interacting Loci

**DOI:** 10.1371/journal.pgen.1003414

**Published:** 2013-03-28

**Authors:** Lin Wang, Wei Zheng, Hongyu Zhao, Minghua Deng

**Affiliations:** 1 Center for Quantitative Biology, Academy for Advanced Interdisciplinary Studies, Peking University, Beijing, China; 2 Department of Biostatistics, Yale School of Public Health, New Haven, Connecticut, United States of America; 3 LMAM, School of Mathematical Sciences, Peking University, Beijing, China; 4 Center for Statistical Science, Peking University, Beijing, China; Princeton University, United States of America

## Abstract

Expression quantitative trait loci (eQTL) studies have generated large amounts of data in different organisms. The analyses of these data have led to many novel findings and biological insights on expression regulations. However, the role of epistasis in the joint regulation of multiple genes has not been explored. This is largely due to the computational complexity involved when multiple traits are simultaneously considered against multiple markers if an exhaustive search strategy is adopted. In this article, we propose a computationally feasible approach to identify pairs of chromosomal regions that interact to regulate co-expression patterns of pairs of genes. Our approach is built on a bivariate model whose covariance matrix depends on the joint genotypes at the candidate loci. We also propose a filtering process to reduce the computational burden. When we applied our method to a yeast eQTL dataset profiled under both the glucose and ethanol conditions, we identified a total of 225 and 224 modules, with each module consisting of two genes and two eQTLs where the two eQTLs epistatically regulate the co-expression patterns of the two genes. We found that many of these modules have biological interpretations. Under the glucose condition, ribosome biogenesis was co-regulated with the signaling and carbohydrate catabolic processes, whereas silencing and aging related genes were co-regulated under the ethanol condition with the eQTLs containing genes involved in oxidative stress response process.

## Author Summary

eQTL studies collect both gene expression and genotype data, and they are highly informative as to how genes regulate expressions. Although much progress has been made in the analysis of such data, most studies have considered one marker at a time. As a result, those markers with weak marginal yet strong interactive effects may not be inferred from these single-marker-based analyses. In this article, using joint expression patterns between two genes (versus one gene) as the primary phenotype, we propose a novel statistical method to conduct an exhaustive search for joint marker analysis. When our method is applied to a well-studied dataset, we were able to identify many novel features that were overlooked by existing methods. Our general strategy has general applicability to other scientific problems.

## Introduction

eQTL studies aim to uncover the genetic architecture underlying expression regulation. In the past decade, they have been conducted in many organisms, including yeast, drosophila, mouse, human and many others [1]–[5]. A common approach in eQTL data analysis is to consider association between each expression trait and each genetic marker through regression analysis, and attention is usually focused on those trait-marker pairs whose associations are significant after multiple comparison adjustments. Despite great success with this approach, some regulatory signals may not be detected due to the complex nature of regulatory networks. For example, genetic buffering relationships often exist in the phosphorylation regulatory network in yeast [6], where pairs of regulators have overlap in function. Similar phenomenon has also been observed in the transcriptional regulatory network in yeast [7]. Single marker analysis may not capture such regulatory patterns, where the genetic effects act through interactions between markers, necessitating the need to incorporate interactions in the analysis. However, extending beyond single marker analysis presents many challenges including the computational demand and the lack of statistical power, because a much larger number of models need to be considered and the need to control the overall false positive results. Storey *et al.* [8] developed a step-wise regression method to detect epistasis on the genome-wide scale. This method is computationally feasible but may miss epistatic effects involving markers having weak marginal effects. Wei *et al.* proposed a Bayesian partition model which may detect more loci having epistatic effects but weak marginal signals [9]. However, this Bayesian approach did not compare favorably with an exhaustive search scheme to detect features with weak marginal signals but strong epistatic effects in practice [10]. To reduce the model search space and increase statistical power, Lee *et al.* adopted genetic interaction networks identified by large-scale synthetic genetic array (SGA) analysis as prior for detecting epistasis in yeast [11]. Since they only consider interacting SNPs that have already been identified, its application is limited to those organisms where comprehensive prior knowledge is available, which is rare in practice.

Although most eQTL studies considered the expression levels of individual genes as response, a conceptually different approach was proposed by Li *et al.* [12] to consider “liquid association” (LA) between a pair of genes. LA aims to identify differential co-expressions, versus differential expressions, across different samples/conditions and the identified LA may offer insights that may not be captured by analysis based on single genes. Li and colleagues later introduced this 2D-trait concept into eQTL study [13]. The goal of such 2D-trait based eQTL analysis is to identify genetic markers that can affect the co-expression patterns between two genes. Since co-expression patterns reflect co-regulation status, such 2D-trait analysis can assess whether the co-regulatory relationship between two genes is associated with certain genetic markers, which is complementary to analyzing the expression patterns of individual genes. For example, in signal transduction pathways, transcriptional factors (TFs) are often regulated by post-transcriptional regulation such as phosphorylation and dephosphorylation. Such regulations are difficult to detect because there may be little change at the expression levels for these genes. However, post-transcriptional regulation does affect TFs' activities, which further affect the expression levels of their target genes. In this case, if a genetic marker affects post-transcriptional regulation, its effect may be captured by the change of co-expression patterns of the targets of TFs, so a LA analysis may lead to the identification of such markers, where it may be difficult to detect these signals using single gene expressions as the response. Recently, Ho *et al.* [14] proposed a conditional bi-variate normal model to analyze LA that simultaneously captures means, variances, and correlation between a pair of genes. Under a similar framework, Chen *et al.* [15] proposed a penalized likelihood approach to effectively detecting causal genetic loci using iterative reweighted least squares, and Daye *et al.* [16] further considered the heteroscedastic problem. Although these methods have broadened the scope of eQTL analysis, none have considered the possibility that markers may have no or weak marginal effects but strongly interact to affect the correlations patterns among gene expressions, which may happen if there is genetic buffering between the markers and this is the focus of our current manuscript.

One major challenge to consider interactions effects on 2D-traits is the large number of models to be examined. For example, with 6000 genes in yeast, a total of 18 million 2D-traits can be formed. If we collect 4000 markers from each yeast strain, considering each pair of markers for their interaction effects will involve 8 million pairs of markers. Therefore, an exhaustive search of all 2D traits versus all marker pairs will evaluate 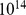 models, a prohibitive number with the current computing power based on the existing methods mentioned above. In this manuscript, we propose a computationally efficient algorithm to identify these Epistasis-2D associations based on conditional bivariate models and likelihood ratio test. In our procedure, we proposed to use a statistic called PA (Potential of Association) to filter out trait and marker sets that are unlikely to be significant before performing the more rigorous likelihood ratio tests. When we applied our method to a yeast eQTL dataset, we were able to identify many “Epistasis-2D” associations that could not inferred from single marker based analysis, where 2D refers to our focus on gene co-expression patterns and epistasis refers to our focus on detecting how loci interact to affect 2D-traits.

## Results

### Detecting Epistasis-2D associations

#### Overview of our strategy

**Figure 1:**
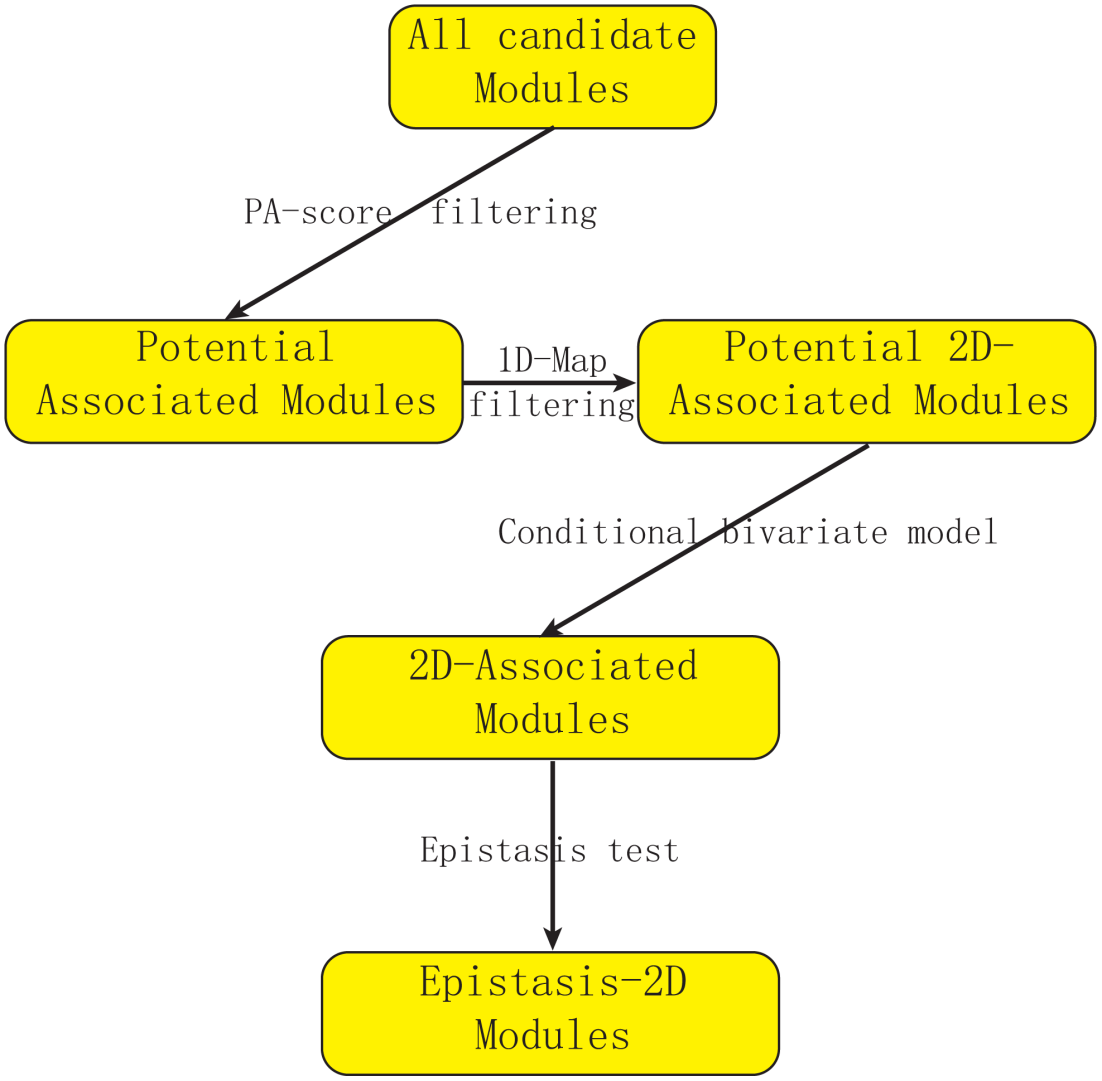
Flowchart of our strategy for detecting epistasis based on 2D-trait. We first use PA-score to filter out modules unlikely to be significant. Then we filter out modules where the association can also be detected using expression levels (1D-traits). We introduce a conditional bivariate model to characterize Epistasis-2D module and use the likelihood ratio test to define p-value. Finally, we perform an epistasis test to remove modules with only marginal signals (Details in Text S1).

Figure 1 describes our strategy to detect Epistasis-2D associations. In this manuscript, we define a module as the collection of a pair of loci and a pair of genes, and our objective is to find Epistasis-2D modules where the two loci interact to affect the co-expression patterns between the two genes in the module. To facilitate statistical analysis, the joint conditional distribution of the two genes for a given pair of genotypes at the two loci is modeled as a bivariate normal distribution, where we are primarily interested in whether the correlation between two genes is dependent on the joint genotypes between two markers. Under the null hypothesis, all the conditional correlations are the same, whereas they differ under the alternative hypothesis. We used the likelihood ratio test to test the null hypothesis. Because it is computationally prohibitive to consider all possible modules using the likelihood approach, we employed a statistic called PA (Potential of Association) to filter out modules unlikely having an association signal. Due to our focus on 2D-traits, we are not interested in those modules containing linkages that can be identified using 1D-traits in this manuscript. Neither are we interested in those modules having only marginal signals.

#### Conditional bivariate models

Traditionally, for a given phenotype, the genetic effects of two loci are often modeled as
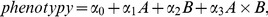
(1)where 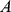 and 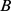 are the coded genotypes at the two loci, 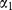 and 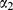 represent the effects of marker 1 and marker 2, and 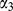 corresponds to the interaction effect between markers 1 and 2. Under this model, the presence of epistasis is captured by a non-zero 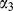 term. Now for a pair of phenotypes, their bivariate phenotypes (X,Y) can be modeled by a bivariate normal distribution [15],
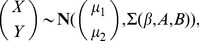
(2)where 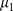 and 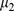 are the mean values of X and Y, which are set as constant in our model.
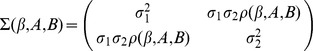
is the covariance matrix and 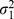 and 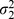 are the variances for the two traits, respectively. As discussed above, our main interest in this paper is the co-expression between two genes, i.e. the correlation term in the matrix 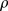 which we model in the following form:
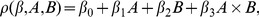
(3)where 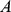 and 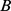 were defined before and 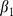, 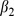, and 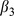 have similar interpretations as 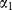, 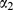, and 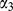 but quantify the marker effects on the correlation.

We note that there is an extensive literature on the difference between statistical interactions and biological interactions because the presence or absence of statistical interactions depends on the specific statistical models used and the scale of the response variable. When each marker has two genotypes (coded by 0 and 1), models (1) and (3) fully parameterize the relationship between phenotype and the four possible genotypes, namely (1,1), (1,0), (0,1) and (0,0) [8]. In this case, we can reformulate model (3) as
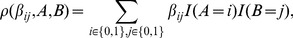
(4)where I is the indicator function, i.e. I(A = i) = 1 if A = i and 0 otherwise.

**Figure 2:**
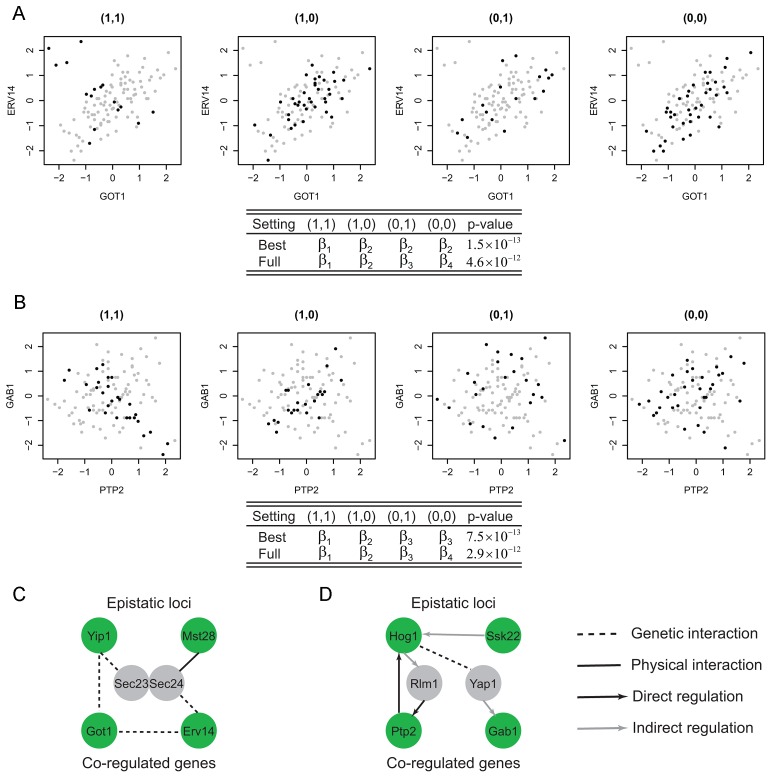
Epistasis-2D modules. In module (A), the co-expression patterns between the two genes *GOT1* and *ERV14* depend on the joint genotype of the two loci. For samples with genotype (1,1) the co-expression correlation is different from the other samples. Hence the proper parameter setting in the conditional bivariate model is (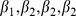) where only two parameters are required. Similarly, in module (B), the co-expression patterns between the two genes *PTP2* and *GAB1* can be classified to three categories with the proper parameter setting being (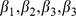). (C) The two candidates in module A, Yip1 and Mst28, may interact to regulate *GOT1* and *ERV14* through the mediator complex Sec23/Sec24. (D) The two candidates in module B, Hog1 and Ssk22, may interact to regulate *PTP2* and *GAB1* through two TFs Rlm1 and Yap1.

Although we could use this most general model to identify interesting modules, a model with fewer parameters may be preferred to achieve a balance between the goodness of fit and parsimony of the model. For example, Figure 2A–2B illustrate two examples where fewer than four parameters are needed to model the co-expression pattern. In Figure 2A, the correlation between the two genes for samples having genotype (1,1) (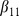) is different from the samples with other genotypes. Hence two instead of four parameters are needed to model this module. Similarly, three parameters are needed for the example shown in Figure 2B where samples having genotypes (0,1) or (0,0) have uncorrelated phenotypes. For the best fitted parameter settings with two or three parameters, the p-values of these two example are 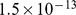 and 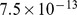, whereas those for the full model are 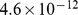 and 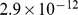. In this article, we test all possible parameter settings for each module and select the model with the most significant p-value. This approach may yield simpler interpretations of the modeling results when fewer parameters are used. In addition, more modules were identified at the same false discovery rate control using our approach compared to the approach based on the full model (Materials and Methods, Text S1).

#### Filtering step to reduce computation burden

There is a computational barrier to directly apply our model to large scale data because estimating parameters in the conditional bivariate models needs numerical solution through iteration [15]. To reduce computation burden, we propose a filtering step which removes modules with low possibility to be significant from further considerations using a statistic called “PA” (Potential of Association). This statistic estimates the lower bound of the p-value for each module. Because PA can be directly calculated from the observed values (expression values and genotypes) without numerical iterations, it can be used to remove most modules before applying the conditional bivariate model. Our real data analysis showed an average reduction of 16 fold computational time with this filtering step (Materials and Methods, Text S1).

### Application to yeast eQTL data

We applied our method to a dataset containing gene expressions and genotypes for 109 segregants from a cross between laboratory (BY, noted as 1) and wild (RM, noted as 0) strains of *Saccharomyces cerevisiae* [3]. The expression levels were measured under two different conditions: glucose and ethanol. We applied our method to the expression data collected under these conditions, and identified 225 and 224 pairs of genes (2D-traits), respectively, whose correlation patterns were under the epistatic control of pairs of markers at an estimated false discovery rate (FDR) 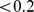 (Materials and Methods, Text S1, Table S3). As far as we are aware, none of these detected marker interactions have been reported to affect expression traits, and our results revealed a new group of regulation patterns that have been overlooked in the literature. Among the 225 and 224 gene pairs, there is an enrichment of pairs having the same functional annotations (31 out of 225 with a p-value of 0.05 and 58 out of 224 with a p-value of 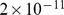) according to GO slim. Despite this statistically significant enrichment, most pairs have different functional annotations suggesting either unknown functions for these genes or interactions between different biology processes.

We observed that the functional distributions of the Epistasis-2D associations are dependent on the environment condition under which the eQTL data were collected. This is consistent with the literature on the importance of the environment on gene expression regulations [17]. Also consistent with previous finding that the trans-acting linkages differ under different environmental conditions [18], our results suggest that trans-acting loci are related to the environment related stress response pathways. The modules identified by our method may be followed up with experimental studies for validation and learning to gain further insights on their biological relevance.

#### Examples of Epistasis-2D modules

Among the Epistasis-2D modules (Table S3) identified by our method, many are biologically meaningful. For example, Figure 2A shows a module detected under the glucose condition where the two genes (*GOT1* and *ERV14*) are functional in ER to Golgi vesicle-mediated transport, whereas the two markers interacting with each other to affect the co-expression patterns between these two genes are located at chromosome VII:833786-858604 and chromosome I:187640-193251. These two chromosomal intervals contain 14 and 2 genes, with each having a candidate that also functions in ER to Golgi vesicle-mediated transport: *YIP1* and *MST28* (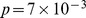, Materials and Methods). Both Yip1 and Mst28 are integral membrane proteins that are involved in COPII transport vesicle formation [19], [20]. Literature suggests synthetic lethality of Yip1 with the heterodimer of the COPII vesicle coat Sec23-Sec24 and physical interaction between Mst28 and Sec23-Sec24 [19], [20]. These experimental results suggest potential interactions between Yip1 and Mst28. In addition, Got1 was identified as a suppressor functioning in the same pathway as Yip1 that regulates biogenesis of COPII vesicle [21]. This observation suggests a regulatory relationship between Yip1 and Got1. Finally, Erv14 is involved in vesicle formation [22] and interacts genetically with both Got1 [23], [24] and Sec23 [25]. In summary, our results and the literature suggest that Yip1 and Mst28 may interact to regulate the co-expression of Got1 and Erv14, and the heterodimer Sec23-Sec24 may mediate their effects (Figure 2C).

Another module is shown in Figure 2B with two genes, *PTP2* and *GAB1*, whose co-expression patterns are epistatically regulated by two loci on chromosomes XII and III. Ptp2 is a phosphatase that dephosphorylates Hog1 in high osmolarity sensing (HOG) mitogen-activated protein kinase (MAPK) pathway. Gab1 is a GPI transamidase subunit and may play a role in the recognition of the attachment signal. The two chromosomal intervals (chromosome XII:370434-388933, chromosome III:240331-264124) contain 8 and 13 genes, and each contains a candidate gene that functions in the HOG MAPK pathway, *HOG1* and *SSK22* (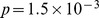, Materials and Methods). Ssk22 is a MAP kinase kinase kinase (MAPKKK) and Hog1 is a MAP kinase (MAPK). *PTP2* is known to be induced by Hog1-dependant transcriptional factor Rlm1 [26]. *GAB1* is transcriptionally regulated by Yap1 [27], [28], which is also the substrate of Hog1 [29]. Hence, the genetic interaction between Hog1 and Ssk2 and their regulation on *PTP2* and *GAB1* is supported by existing literature on the HOG pathway (Figure 2D).

#### Clustering in the epistasis map reveals functional genetic modules

**Figure 3:**
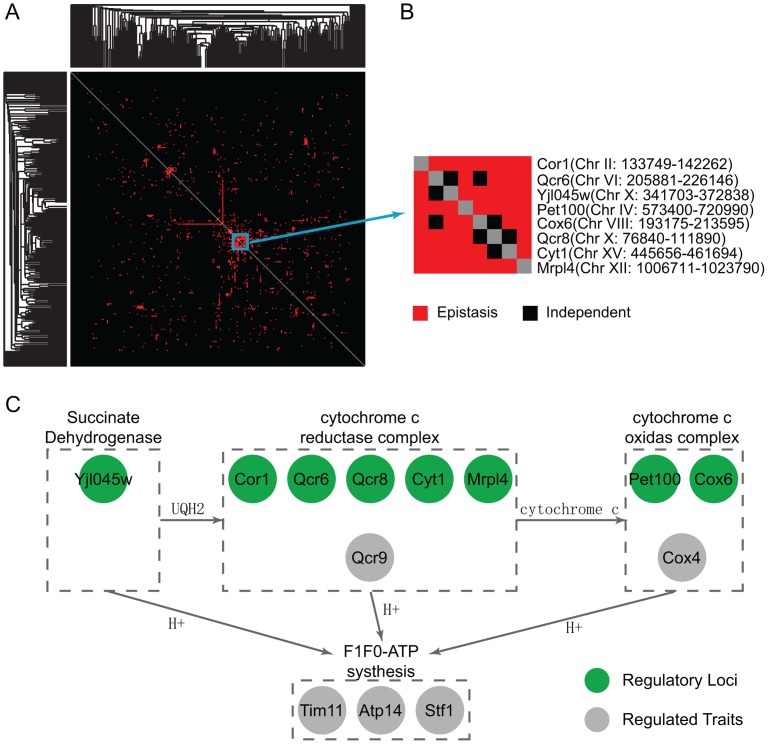
Clustering in the epistasis map reveals a functional genetic module in oxidative phosphorylation pathway. (A) The clustering heatmap of the detected epistasis under the glucose condition. (B) One cluster contains eight highly interacting loci. Their co-targets in the module are mostly functional in the oxidative phosphorylation pathway. There are candidates also functional in the same pathway at these eight loci. (C) A diagram showing the regulatory pathway from the literature, where the green circle represents the candidates and the grey circle represents their targets in the modules.

The locus pairs identified that epistatically interact to regulate the co-expression patterns of gene pairs may be inferred to have genetic interactions and such interactions can be used to develop a global genetic interaction map. We applied the hierarchical clustering to this interaction map and found densely interacting locus clusters (Figure 3A, Materials and Methods). Under the glucose condition, there was one cluster containing eight genetic intervals (Figure 3B). These pairs share similar target gene pairs including six genes (*COX4*, *QCR9*, *ATP14*, *TIM11*, *STF1* and *DBP8*), all except *DBP8* are encoding proteins functional in oxidative phosphorylation, whereas *DBP8* is a ribosomal gene. The expressions of oxidative phosphorylation genes are repressed by glucose, whereas the expression of ribosomal genes are induced by high glucose signal. Therefore, it is plausible that the expression of *DBP8* is correlated with oxidative phosphorylation genes for samples with certain genotypes under glucose condition. The eight intervals are enriched with oxidative phosphorylation candidates (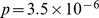, Materials and Methods). More specifically, six intervals contain one candidate annotated to function in oxidative phosphorylation, including *COR1*, *QCR6*, *QCR8*, *CYT1*, *COX6* and *YJL045W*. The other two intervals contain *MRPl4* and *PET100* which participate in the oxidative phosphorylation process although they are not noted in GO. Among the proteins encoded by these candidates, Yjl045w is responsible for the oxidation of succinate and production of ubiquinone, which is the substrate for cytochrome c reductase complex containing Cor1, Qcr6, Qcr8, Cyt1 and the target Qcr9. In addition, Mrp14 is associated with the Cbp3-Cbp6 complex to promote cytochrome c reductase complex synthesis and assembly [30]. The cytochrome c reductase complex oxidizes ubiquinone while reducing cytochrome c, which in turn serves as the substrate for cytochrome c oxidase complex including the candidates Cox6 and the target Cox4. Pet100 is a chaperone that specifically facilitates the assembly of cytochrome c oxidase. During these processes protons are transferred out of the mitochondrial membrane, and back into the mitochondrial matrix. The energy derived from the movement of these protons is used in ATP synthesis, and the targets Atp14, Tim11, Stf1 are functional in the F1F0-ATP synthesis (Figure 3C). Hence, these detected epistatic relationships are well supported by their close connections in the oxidative phosphorylation pathway.

#### Function analysis reflects how environment modulates regulatory modules

**Figure 4:**
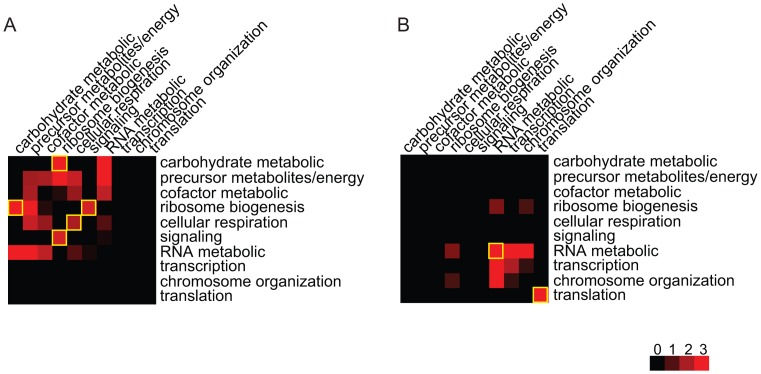
Functional distributions of the gene pairs in the module across different bioprocesses. (A) Under the glucose condition, ribosome biogenesis genes are co-regulated with signaling and carbohydrate metabolic in the Epistasis-2D modules. Genes within cellular respiration are also co-regulated. (B) Under the ethanol condition, genes within RNA metabolic and translation are co-regulated. The values shown in the figure are the −log_10_ (p-value) of the enrichment test for each pair of functions.

To understand how environmental conditions modulate the effects of genetic variants on phenotypic traits, we investigated whether the gene pairs in the inferred Epistasis-2D modules are enriched for certain biological processes. For the 225 and 224 2D-traits identified under the two conditions, Figure 4 summarizes the pairs of functions enriched for co-regulated gene pairs (Materials and Methods). It can be seen that the patterns are quite different between the two conditions. Under the glucose condition, ribosome biogenesis tends to be co-regulated with carbohydrate metabolic process (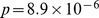) and signaling (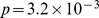). Genes within the cellular respiration process also tend to be co-regulated (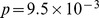) (Figure 4A). Under the ethanol condition, genes within the RNA metabolic process (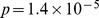) and translation (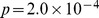) tend to be co-regulated (Figure 4B).

#### Glucose response pathway modulates ribosome-related modules

To understand the differences observed between the two conditions, we investigated the regulatory loci to bridge the gap between environment and co-regulated processes. Ribosome biogenesis is associated with the glucose condition, with ribosome biogenesis genes induced in response to high, but not low, glucose signals [31]. This is consistent with our observation that ribosome biogenesis related regulation was only identified under the glucose condition. In addition, yeast responds to glucose via several glucose-sensing and signaling pathways, two of which are reflected in our results: the main glucose repression pathway through the complex SNF1 complex to inhibit the Mig1 repressor-containing complex, and the Gpr1/Gpa2 glucose-sensing pathway which activates cAMP synthesis [32].

**Figure 5:**
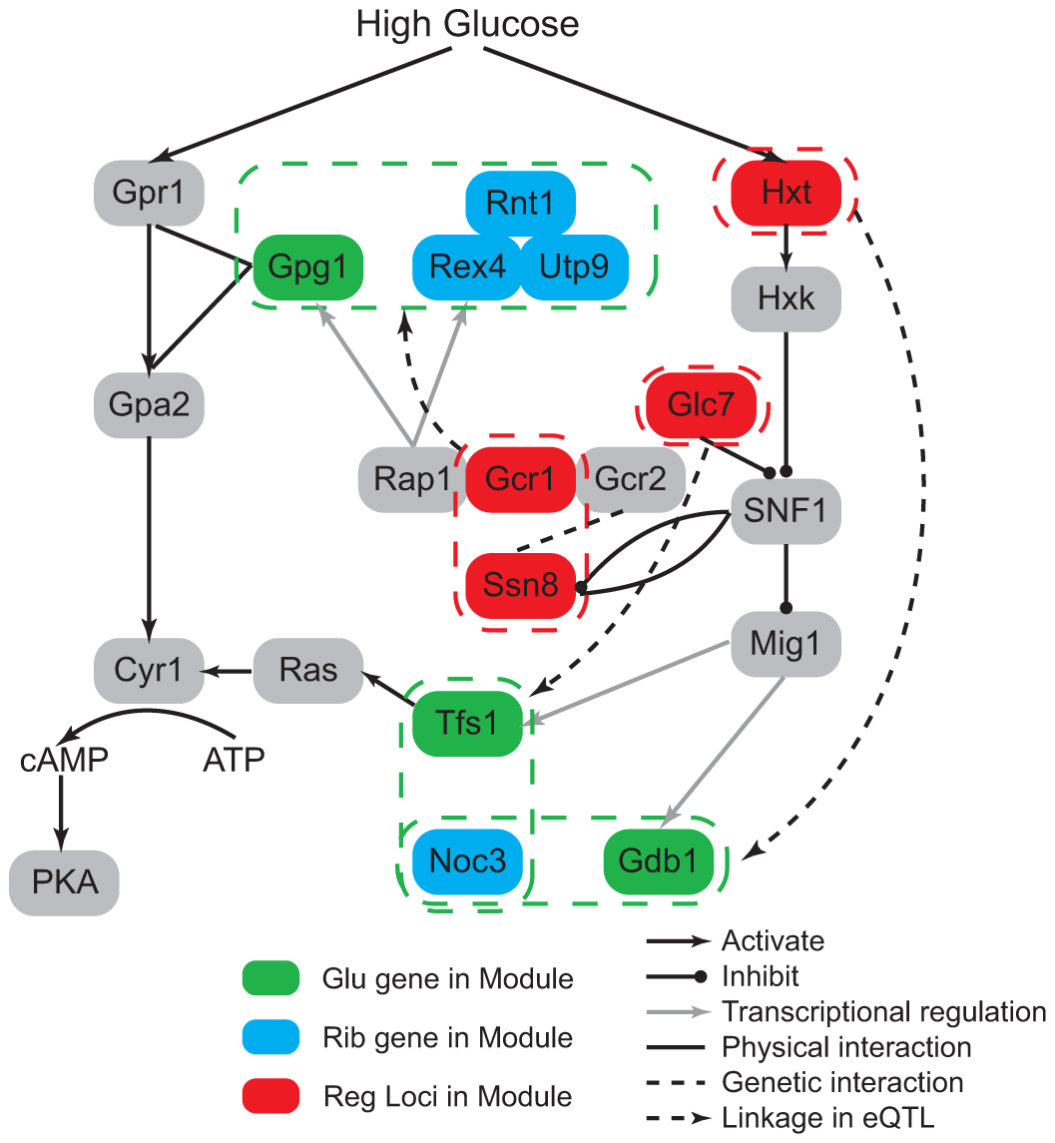
Glucose modulates ribosome-related modules through glucose response pathway. The regulatory loci in the modules contain genes involved in the glucose response pathway (red nodes). This indicates how the glucose condition modulates the co-expression pattern between ribosome genes (blue nodes) and glucose signaling or metabolic genes (green nodes).

To gain insights of this environment modulated regulatory relationship, we first studied the linkage between ribosome biogenesis and signaling process. In our results, two signaling genes (*GPG1* and *TFS1*) were co-regulated with ribosomal genes under the glucose condition. They both encode proteins that function in the glucose signaling pathway. Gpg1 interacts with the glucose sensor Grp1 and Gpa2 [33], and Tfs1 could activate the cAMP/PKA pathway. The regulatory loci of these modules are enriched with glucose metabolic genes (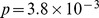, Materials and Methods). *GPG1* is co-regulated with three ribosome genes (*REX4*, *RNT1*, *UTP9*), and associated with two genetic intervals: chromosome XVI:387239-420441 and chromosome XIV:558284-595885. These two chromosomal intervals contain 14 and 16 genes and each contains a candidate that functions in the glucose response process: *GCR1* and *SSN8*. Gcr1 forms a complex with Rap1 and Gcr2 to transcriptionally activate glycolytic genes [34], [35], and Rap1 was detected to be the transcription factor of *GPG1* and *REX4* [27], [28]. *SSN8* encodes the RNA polymerase II holoenzyme and is involved in glucose repression [36], it is repressed by SNF1 complex and also physically interacts with Snf1 (Figure 5). High-throughput study has detected genetic interaction between Gcr2 and Ssn8 [37], which may be related to the epistatic interaction between Gcr1 and Ssn8. How this interaction could influence the regulation of the genes in the modules maybe an interesting direction for future studies. The co-regulated ribosomal gene pairs *TFS1* and *NOC3* are associated with chromosomal intervals: XII:514835-516700 and V:430931-458085. The former interval is located near *TFS1* and the latter contains 11 genes, among which one candidate *GLC7* is a well known regulator in glucose response. Glc7 is the phosphatase that inhibits SNF1 complex (Figure 5). SNF1 complex inhibits the Mig1 complex, which is the transcriptional regulator of *TFS1* [27], [28]. Further more, we can also find glucose response genes among the co-regulatory loci of ribosome biogenesis and carbohydrate metabolic process. For example, one module contains two genes *GDB1* and *NOC3*. *GDB1* encodes a glycogen debranching enzyme and *NOC3* is involved in ribosome biogenesis. Their regulatory loci are located at two chromosomal intervals: IV:1149761-1185630 and XIII:286122-298193, where enriched with glucose transport genes (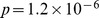, Materials and Methods). The former contains 15 genes and three candidates (*HXT3*, *HXT6*, *HXT7*) encode glucose transporters in glucose response pathway. The latter interval contains two genes and one candidate *HXT2* also encodes a transporter in glucose response pathway (Figure 5).

#### Oxidative stress in ethanol modulates aging-related modules

Under the ethanol condition, 25 pairs of RNA metabolic genes were co-regulated among the 224 identified 2D-traits containing 43 unique genes. We note that 11 genes function in the silencing process, which are known to be related to aging [38], [39]. In addition, two other genes *RAS2* and *MSN2* are known to function in the aging process [40]. In total, 16 of these 25 (64%) pairs contain at least one aging related gene. These aging-related modules and proposed regulatory candidates are shown in Table S5. Among the 11 silencing genes, six form three co-regualted pairs: *UBP10*-*HMLALPHA1*, *SPT23*-*RPD3*, and *ESC2*-*ZDS2*. All the regulatory loci in the first two modules and many regulatory loci in the other aging-related modules contain oxidative stress response related regulators (Table S5), which is interesting because there is a conjecture that oxidative stress could induce aging in ethanol environment [38], [39]. The metabolism of ethanol is assumed to induce aging through increased damage from reactive oxygen species (ROS) produced in oxidative stress response [38], [39], but the exact mechanism is not clear. We discuss the details of these modules in the following to gain some insights on how oxidative stress response pathway regulate aging-related process.

**Figure 6:**
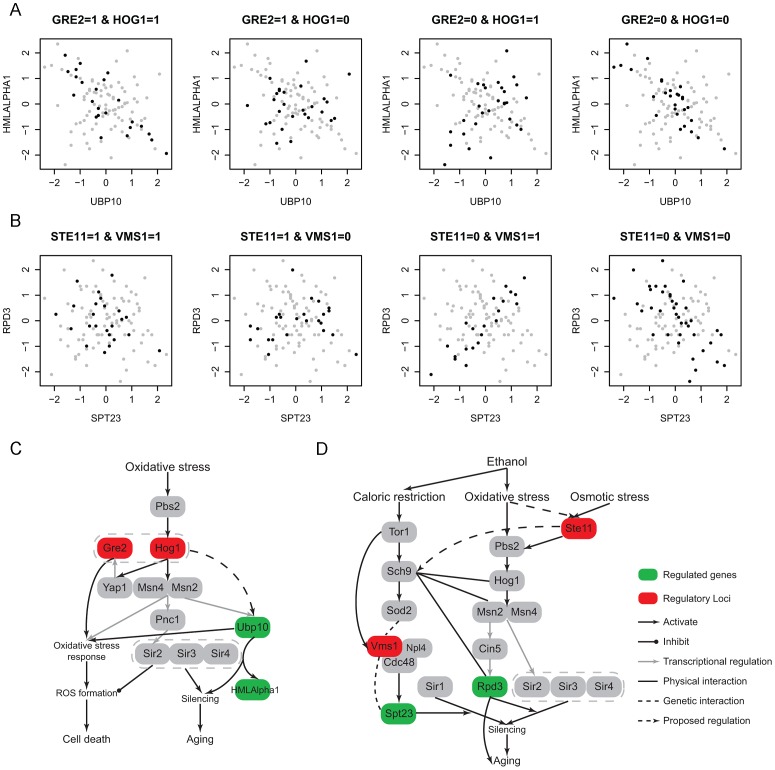
Ethanol modulates aging-related modules through oxidative stress response pathway. (A) One module contains two silencing genes, *UBP10* and *HMLALPHA1*. The regulatory loci contain two oxidative stress response genes, Gre2 and Hog1.; (B) One module contains two silencing genes, *SPT23* and *RPD3*. The regulatory loci contain two oxidative stress response genes, Ste11 and Vms1; (C) A diagram showing the module A related pathway, which indicates a potential regulatory relationship between oxidative stress response and Ubp10 induced silencing process; (D) A diagram showing the module B related pathway. The epistatic interaction between the two candidates Vms1 and Ste11 indicates the potential crosstalk between caloric restriction response and oxidative stress. Moreover, Ste11 may also regulate Pbs2-Hog1 signaling pathway under oxidative stress besides osmotic stress response.

In the *UBP10*-*HMLALPHA1* module (Figure 6A, 6C), Ubp10 is functional in silencing at telomeres and mating-type loci. It cooperates with Sir4 to regulate the expression of subtelomeric genes and mating-type silencing related genes including HMLALPHA1. The mutant of Ubp10 also reveals its influence in the oxidative stress response [41]. Sir4 is a member of the chromatin silencing complex (Sir1-4) which is the regulator involved in assembly of silencing complex at telomeres and mating-type loci [42]. The SIR complex is also known to link silencing and aging [43], [44]. The regulatory loci in this module are located at chromosome XV:37207-44482 and chromosome XII:370434-388933 which contain two and eight genes, respectively. Only one gene *GRE2* in the former interval is noted to have a function. *GRE2* is involved in the oxidative stress response and regulated by the HOG pathway. The latter interval contain the candidate *HOG1* which are also involved in oxidative stress response (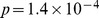, Materials and Methods). Hog1 is a kinase that regulates several transcription factors including Msn2,4 and Yap1 to respond to oxidative stress, and Gre2 is known to be regulated by Hog1 through Yap1 and function in the repair of oxidative damage although the details of its role are still not clear [45]. In addition, the expression of *UBP10* is affected by mutations in Msn2 [27], [28] which may mediate the regulation between Hog1 and Ubp10. A recent study indicates that Hog1 could activate Sir2 through Msn2,4 to suppress Hog1 induced ROS accumulation, but this regulation does not involve Sir4 indicating that the function of Sir2 here does not depend on its function in silencing [46]. This observation illustrates a partial interaction between oxidative stress response and silencing process downstream. Our results suggest that the function of Ubp10 in silencing is regulated by Hog1 and Gre2, which builds the upstream linkage between oxidative stress and silencing regulation. This observation indicates that oxidative stress response and silencing may be jointly regulated. The epistatic interaction between Hog1 and Gre2 needs to be experimentally validated to further characterize regulation mechanism.

In the *SPT23*-*RPD3* module (Figure 6B, 6D), Rpd3 is a histone deacetylase that is involved in Sir2-mediated silencing [47] and aging [48]. Spt23 may affect silencing caused by Sir1, but its role in silencing is not clear [49]. Their regulatory loci (chromosome XII:849485-851826 and chromosome IV:527445-555043) contain 1 and 11 genes, respectively. The former interval contains only one gene *STE11*, which is the signal transductor in the HOG pathway responding to high osmolarity (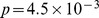, Materials and Methods). *RPD3* is transcriptionally regulated by Cin5 [27], [28], which is known to be regulated in HOG pathway through Msn2 under osmotic stress [50]. Rpd3 also functions with Hog1 and Msn2 in the same pathway to activate osmo-responsive genes. Although these all indicate regulatory relationships between the HOG pathway and Rpd3 under osmotic stress, the regulation may also exist under oxidative stress since many HOG pathway genes are also involved in oxidative stress response such as Hog1, Msn2,4 and Cin5. To our knowledge, the role of Ste11 in oxidative stress response is not clear, which maybe an interesting direction for future studies. The later interval contains one candidate *VMS1* which also functions in oxidative stress related process. Vms1 forms a complex with Cdc48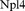 to regulate targets under oxidative stress [51], [52], while Cdc48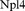 modulates Spt23 through direct binding [53]. Although the latter regulation is not discovered under oxidative stress and it is not clear whether this regulation is related to the function of Spt23 in silencing, these known regulations provide a potential framework to interpret the regulation between Vms1 and Spt23. In addition, high-throughput experiments have captured the genetic interaction between Spt23 and Vms1 [7], [54]. To understand the epistatic interaction between Vms1 and HOG pathway, we investigated the upstream regulators of Vms1. Under oxidative stress Vms1 is genetically interacted with Sod2 [51]. Sod2 is downstream of Sch9, and Sch9 is regulated by Tor1. Tor1 is the main kinase in TOR pathway that is well known to regulate aging under caloric restriction [38]. *VMS1* was also found to be regulated by Tor1 [55]. From observing the co-expression pattern in the module (Figure 6B), we note that only when the genotype of Ste11 is 0 (wild RM), *SPT23* and *RPD3* could be co-regulated, moreover the sign of their expression correlation depends on the genotype of Vms1. It seems that Ste11 provides the signal upstream of Vms1. In addition, the regulation of *VMS1* in this module also exists under oxidative stress. Hence, there may be some crosstalk between oxidative stress and caloric restriction response pathways. This is plausible because the two stresses could both be induced by ethanol environment and cause silencing and aging. Interestingly, Huang *et al*. [56] recently demonstrated that Sch9 could integrate nutrient signals from the TOR pathway and oxidative stress signals from sphingolipids to regulate aging. Although their proposed signal transduction from oxidative stress to Sch9 was not related to the HOG pathway, other studies have shown the close interactions between Sch9 with HOG-related genes like Hog1, Gre2, Msn2,4 and Rpd3 in other processes [57]–[59]. Combined with our results, it is possible that Sch9 could integrate oxidative stress signal from HOG pathway to regulate aging related processes under the ethanol condition.

#### Comparisons between our method and other methods

In this section, we compare the results from our method to those from two other methods: a forward search strategy to identify Epistasis-2D modules (instead of the exhaustive search scheme adopted by us) and an analysis focusing on 1D (instead of 2D) traits. For the forward search method, we extend a forward search algorithm proposed by Storey *et al.* [8] to consider 2D-traits as follows:

- For each pair of traits, identify the locus primarily associated with the co-expression patterns where the correlation between the two traits is modeled as 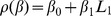, that is, we first identify single loci affecting the changes in correlations between two traits.
- After the identification of the first locus, we conduct the forward search to identify other loci that interacts with the first locus to affect the correlations between the two traits using the conditional bivariate test described above for our method. We also test all possible interaction models for each candidate module with two genes and two loci and select the model with the most significant p-value.

Because our method is based on an exhaustive search scheme, we only focused on the modules identified by our method as the others are not as significant as these identified ones. When we re-scanned the detected 225 and 224 2D-traits using the forward search, only 139 and 112 can be identified to be associated with two loci at the same statistical significance level (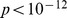) in our method. This is because that the 2D-traits defined by the two genes in each module are linked to interacting loci with weak marginal effects, which were missed by the forward search scheme.

**Figure 7:**
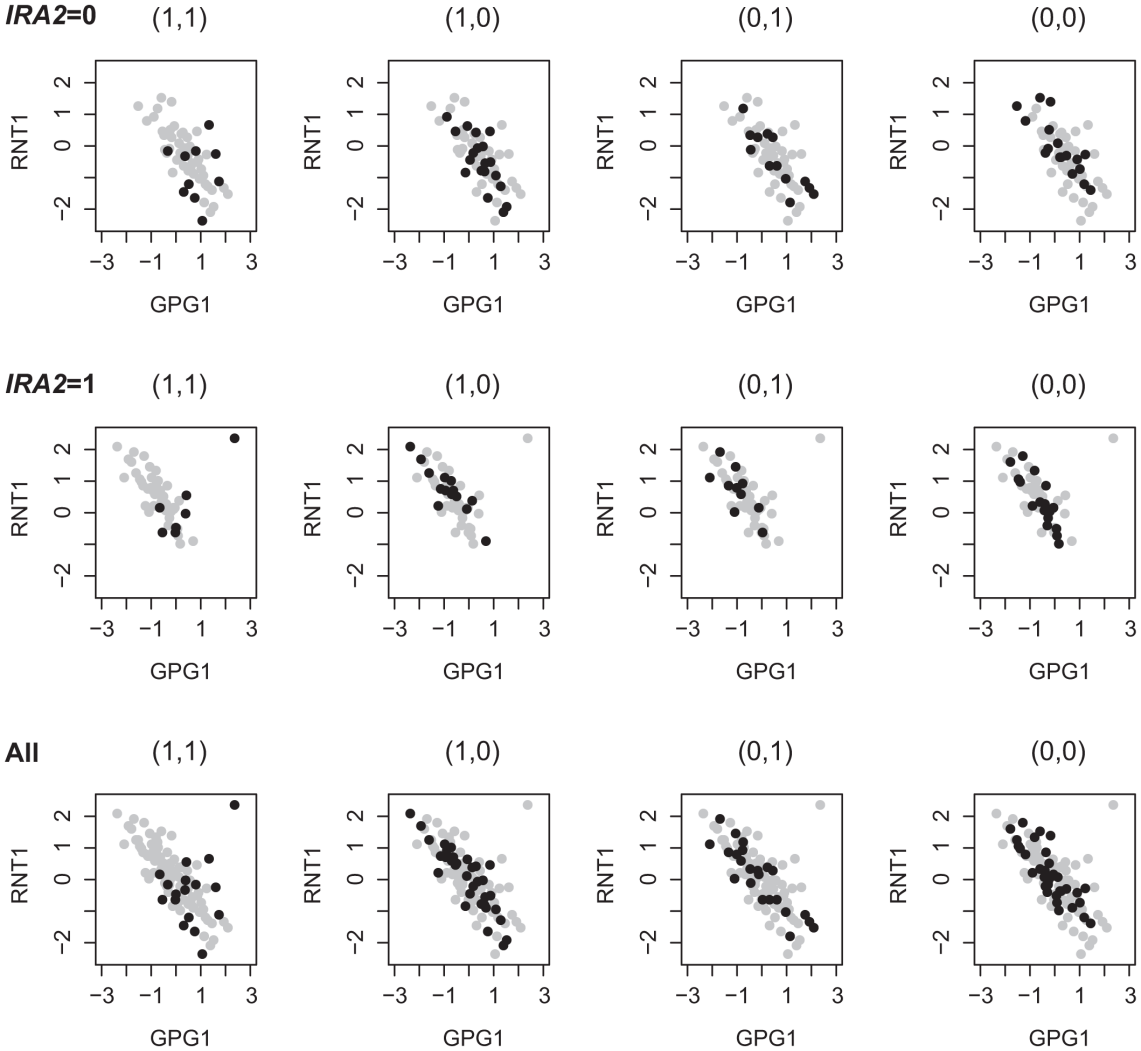
1D-trait and 2D-trait reflect different genetic regulations. *RNT1* is up-regulated in segregants bearing the BY allele at *IRA2* (*IRA2* = 1); *GPG1* is up-regulated in segregants bearing the RM allele at *IRA2* (*IRA2* = 0); the expressions of *RNT1* and *GPG1* are negative correlated except in segregants bearing the BY allele at two other loci containing candidates *GCR1* and *SSN8*.

To investigate the different signals identified from studying 2D-traits versus 1D-traits, we compared the linkage results based on the analysis of single traits for genes involved in the Epistasis-2D modules. Among the 225 and 224 2D-traits, there are 319 and 378 unique genes, respectively. For each gene, we performed the Wilcoxon rank sum test to detect eQTLs in the genome. In total, 135 and 60 genes were found to be linked to at least one locus at the 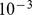 statistical significance threshold. Note that our method excludes all loci that can be found by marginal analysis, so the loci inferred in this single trait analysis have little overlap with the loci identified by our method. We observed three hotspot regions in these eQTLs: hotspot1 (chromosome XV:141621-174364), hotspot2 (chromosome XIV:449639-486861) and hotspot3 (chromosome III:201166-201167). The hotspot1 region is linked to 117 and 9 genes, and Smith *et al.* have identified *IRA2* as the candidate gene in this region to affect energy metabolism and growth related genes [3]. The hotspot2 region is linked to 4 and 8 genes, and Kang *et al.* suggested *RAS2* as the candidate gene in this region to affect gene expressions by perturbing the RAS signal transduction pathway [60]. The hotspot3 region is linked to 4 genes under ethanol condition, and Brem *et al.* have identified *MAT* as the candidate gene in this region to affect mating response related genes [2]. Beside these three regions, no other loci are linked to more than two traits. For those traits not linked to these hotspot regions, 14 and 39 genes, respectively, most (11/14 and 26/39) are cis-linked (the QTLs are located within 10 kb of the traits). Our results suggest that most trans regulated 1D-traits are linked to regulatory hotspots, which tend to affect multiple genes [60]. For example, the hotspot1 region was associated with 1159 and 410 genes under the two conditions at 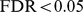 [8]. For genes with eQTLs mapped to the same region, the analysis of their co-expression patterns may identify additional regulators of these genes. For example, Figure 7 shows an example where the expressions of the two genes, *GPG1* and *RNT1*, were both affected by hotspot1. We can see that their co-expressions were regulated by two other markers located at chromosome XIV:558284-595885 and chromosome XVI:368296-408883. The candidate *IRA2* involved in hotspot1 mediates glucose response via the cAMP-dependent pathway. We found candidates *SSN8* and *GCR1* in chromosome XIV:558284-595885 and chromosome XVI:368296-408883 which also function in glucose response as introduced above. This observation suggests that different forms of regulation may exist. Ira2 globally regulates gene expressions in the glucose response related pathways including *GPG1* and *RNT1* [8], whereas Ssn8 and Gcr1 specifically regulate the co-expression between *GPG1* and *RNT1*. Cis-acting eQTL may affect the gene expression through affecting transcription factor binding [61], [62]. Additional association signals besides cis-acting loci suggest other regulatory mechanisms for these genes. Hence, analysis based on 1D-traits and 2D-traits complement each other in identifying regulatory signals and they may reflect different regulation mechanisms.

## Discussion

We have developed a novel statistical approach to identifying gene pairs whose co-expression patterns are jointly regulated by interacting loci through the analysis of eQTL data. Our approach is based on modeling the joint expression levels with a bivariate normal distribution whose covariance matrix is dependent on the joint genotypes at two candidate loci. Although different model search strategies have been proposed to jointly analyze multiple markers and their interactions based on genome wide data, e.g. marginal search, forward search and exhaustive search, the ability to conduct an exhaustive search allows us to identify interacting loci with weak marginal effects [63]. To facilitate an exhaustive search of all gene pairs versus locus pairs, we also proposed a filtering process to only focus on those modules that are likely to be statistically significant. This filtering process is one important component of our strategy to reduce the computational burden without reducing statistical power for discoveries. The application of our method to a yeast data set has identified many interacting loci with weak marginal signals which would not have been found without the exhaustive search strategy. Compared to the existing methods to detect epistasis, we considered the 2D-trait, especially their co-expression patterns, as the phenotype. As discussed in the introduction section, using such 2D-traits may help to detect post-transcriptional regulation from the change of expression correlations between downstream genes. As shown with the examples in the results section, we detected many regulatory loci containing candidate genes encoding kinases or phosphatases that regulate the co-expression correlation between the targets of their TF substrates. None of these modules could have been detected using 1D-traits. Since we only focus on modules which can not be detect using 1D-mapping in this paper, we may miss potential Epistasis-2D modules with genotype-dependent mean values (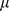) through 1D-Map filtering and assuming 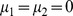 in our model. About 13% modules were filtered using 1D-Map filtering, therefore around 13% Epistasis-2D modules could be missed by our method. Although we could introduce more parameters in our model to allow for genotype-dependent mean values, this may introduce noises that lead to reduced statistical power with limited sample size. More detailed discussion on the trade-off between statistical power and model adequacy is provided in the supplementary materials (Text S1, Figures S6 and S7).

We applied our strategy to a well studied yeast eQTL dataset and detected many epistasis modules, most of which have not been discovered to date and many may be interpreted with existing biological literature. We found that the co-regulated genes in the modules inferred under different environments were enriched for different biological processes. For example, under the glucose condition, ribosome biogenesis tends to be co-regulated with glucose response and glucose metabolic processes. The loci jointly regulating their expression patterns are enriched with genes in the glucose response pathway. Under the ethanol condition, silencing and aging related genes were found to be co-regulated. The loci jointly regulating these genes are enriched with genes in the oxidative response pathway, consistent with the hypothesis that the metabolism of ethanol would induce aging through increased damage from ROS produced in oxidative stress response. Through detailed discussion of several identified modules, we proposed potential regulatory mechanisms between oxidative stress signal and aging process.

Interpretation is difficult in eQTL linkage studies because the detected eQTLs often have low resolution, e.g. large intervals, with many candidate genes. Traditional linkage analysis with single genes and one locus often offers limited information to identify a candidate gene around the locus to understand the linkage signal. Since Epistasis-2D modules detected in our study involve two genes and two loci, the biological association of the two genes offers additional information to prioritize candidate genes in the inferred loci as shown in the examples in the results section. However, significant challenges remain to identify candidate genes in the inferred loci and interpret the results. First, there is genetic buffering in a robust regulatory network, and we may not be able to infer all the direct linkages from eQTL studies. The mediators not observed between indirectly linked loci and genes make it more difficult to interpret the regulatory linkage. Second, in the Epistasis-2D modules, the genetic loci may affect one of the two target genes or both of them, and either situation will cause the variation of the co-expression pattern. This also increases difficulty for interpreting the linkage results. Therefore it is often necessary to incorporate information from other resources to interpret the detected modules. For example, in the oxidative phosphorylation pathway modules we illustrated in the results section, the co-expression patterns between Dbp8 and other oxidative phosphorylation genes are co-regulated. Since the candidates we predicted are all involved in the oxidative phosphorylation pathway, it is quite possible that only the expression of oxidative phosphorylation genes in Dbp8-related modules, but not the expression of Dbp8, is actually affected. Similarly, we also investigated different types of databases to collect evidences and interactions for interpreting other discussed modules. It is important to integrate other data sources including protein interactions, transcription and proteomics data under a consistent framework to better interpret the results. This generic idea has been formalized in different ways for interpreting one-to-one linkages [64]–[67], and more work is needed to adapt these methods to interpret the modules identified by our method. Utilizing our results through integration of multiple data sources is an interesting future direction. Our strategy could also be applied to other eQTL data in mouse or human. For example, in the mouse eQTL data, there are around 2000 markers which is comparable to the yeast data and the number of differentially expressed transcripts was around 8000 [4]. In this case, the search space is on the order of 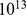, which can be readily handle by paralleling our algorithm. In the human eQTL data, up to over 5,000,000 SNPs may be genotyped and up to 50,000 transcripts may be profiled. This will dramatically increase the computation time. We may reduce the computational burden by focusing only on those transcripts of interest (e.g. those known to be relate to diseases) or setting more stringent cutoffs in the filtering process to accelerate the processing. However, more computationally efficient methods need to be developed to identify Epistasis-2D modules for these data if we want to consider all the traits and markers. One possible direction is to jointly consider multiple markers within a region as those done for GWAS data [68], [69].

## Materials and Methods

### Conditional bivariate model

We define a module in this manuscript as the collection of a pair of loci and a pair of genes, denoted as 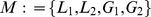, where 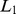 and 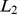 represent two loci and 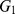 and 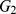 represent two genes. Our objective is to identify Epistasis-2D modules where 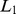 and 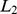 interact to affect the co-expression patterns of 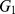 and 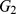. To formally describe our model, we use 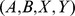 to denote the genotypes of 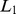 and 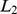 and the expressions of 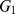 and 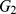. We assume that,
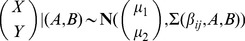
(5)where
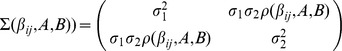
is the covariance matrix, and
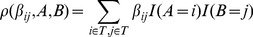
(6)where I is the indicator function, i.e. I(A = i) = 1 if A = i and 0 otherwise, and T is the set of genotypes. For example, in the yeast dataset 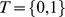. In this study, we focus on associations that can not be detected using 1D-trait (expression level), i.e. we assume that 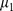 and 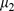 are independent of 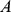 and 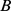. This simple model may have overall good statistical power to detect Epistasis-2D modules as discussed in detail in the supplementary materials (Text S1, Figures S6 and S7). Without loss of generality, we let 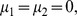 which approximately hold after applying the following transformation to the expression data:

- For each gene, calculate the rank of the expression for each sample, denoted as 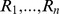;
- Calculate the transformed expression level for each gene as 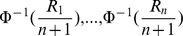, where 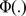 is the cumulative normal distribution;

The normal quantile transformation based on individual genes is a means to “normalize” the sample observations so that our procedure is robust to the effects of extreme observations and/or highly skewed distributions [12], [15] (Text S1, Figures S1 and S2). In our model, we assume that 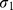 and 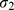 are independent of the genotype because we found this specification achieved a good balance between model adequacy and simplicity. We illustrate this through the analysis of simulated data and a subset of the real data in the supplementary materials (Text S1; Figures S3, S4, S5). We found that although it was feasible to fully consider genotype dependent variances, there may be overall power loss due to additional model parameters, especially when the sample size is limited.

Considering a sample with n individuals, let 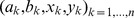 represent the genotypes and expressions in the kth sample. The model parameters 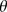 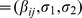 in (8) can be estimated using the maximum likelihood estimates (MLE), where the log-likelihood function is,
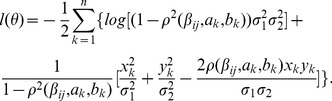
(7)


Our goal is to identify gene pairs whose correlations depends on the joint genotypes of the two loci. We consider 12 epistatic models (Table S1) versus the null hypothesis that the correlation is the same for different joint genotypes. To focus on epistatic interactions, we then compare the most significant model with two single association models. The comparisons are based on the likelihood ratio (LR) test.

### Filtering process

Since MLE needs to solve a numerical optimization problem, applying the tests above to all possible modules is computationally expensive. Therefore, we introduce a statistic “PA-score” (Potential of Association) to estimate the lower bound of the p-value for each module. The PA-score is defined as,
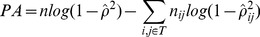
(8)where 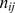 is the number of individuals with genotypes 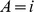 and 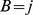, 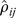 is the Pearson correlation coefficient of the expression levels among the 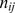 individuals and 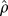 is the correlation coefficient among all the individuals.

We prove in the Text S1 that the expectation of PA corresponds to the lower bound of p-value for each module. In this case, we could control the sensitivity by choosing the cutoff for PA to filter out modules before performing the LR tests. In this paper, we chose a cutoff value of 45 for PA which has an estimated sensitivity 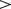
0.995. The sensitivity here is one minus the percentage of the significant LR test modules which will be filtered out by PA score filtering. The details of the sensitivity estimation are provided in the supplementary materials (Text S1, Figure S8). After the PA-score filtering, more than 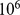 modules remained for each condition. Using the LR tests, we identified 225 and 224 2D-traits whose correlation patterns were under the epistatic control of pairs of markers. Therefore we estimated that 
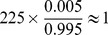
 epistatic controlled 2D-traits was filtered out by PA score in each condition. Since PA can be directly calculated from the data, the filtering process can reduce the total computing time by a factor of 16 from our experiments with the data (Text S1).

### Yeast dataset

We analyzed the yeast dataset collected by Kruglyak and colleagues [3]. The expression data were downloaded from http://www.plosbiology.org/article/info%3Adoi%2F10.1371%2Fjournal.pbio.0060083, with 4,482 genes measured in 109 segregants derived from a cross between BY and RM. The experiments were performed under two conditions, glucose and ethanol. We removed 63 genes with more than 10 missing values in either condition for a total of 4,419 genes analyzed. The authors provided genotypes at 2,956 loci. We combined neighboring loci having fewer than 5 discordant calls among the 109 samples, leading to 820 merged markers. In this manuscript, we still call these merged markers as markers to simplify the discussion. For each marker pair, an individual can have four joint genotypes, (0,0), (0,1), (1,0), and (1,1). We only considered marker pairs where there were at least 15 individuals for each joint genotype. There were a total of 305,301 such pairs. Therefore, we tested 305301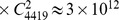 modules. The algorithm was implemented in R. Applying our procedure to one condition took one week of one CPU on a Linux cluster with 2.40 GHz CPU.

### False discovery rate estimation

We estimate the false discovery rate (FDR) through a permutation technique similar to previous study [8]. Specifically, we randomly permutated the expression data across all the genes and applied our procedure to the permuted data set using exactly the same setting as the real dataset. That is we used the same cutoff 45 for PA, and the same cutoff 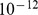 for p-values of the LR tests (also select the best model). For a given threshold for LR tests, we counted the number of unique 2D-traits involved in modules with their p-values lower than the threshold. Note that we did not use the number of modules to calculate FDR because a 2D-trait may be mapped to multiple neighboring marker pairs since neighboring markers tended to have similar genotypes. Hence, we use 2D-trait to label the modules for FDR estimation. We performed ten permutations for each condition to yield ten sets of 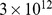 simulated null modules, and the results were consistent across these ten permuted data sets (Table S2). For example, at the threshold value of 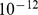, the average number of unique 2D-traits involved in modules with a statistical significance level less than 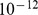 in the permuted dataset was 36.4 (SD = 4.9) and 38.5 (SD = 5.1), respectively. Therefore, with a total of 225 and 224 significant 2D-traits identified for the observed data under the two conditions, the estimated FDR was 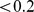 for both conditions (Text S1, Table S3).

### Merging of Epistasis-2D modules

Among the inferred Epistasis-2D modules, neighboring markers tended to show similar patterns of interactions as discussed previously [70]. We merged neighboring markers with fewer than 15 individuals showing discordant genotypes among all samples, leading to 266 merged markers for clustering analysis. Table S4 listed all detected Epistasis-2D modules after the merging.

### Clustering in the epistasis map

We define an epistasis map E under a specific condition as
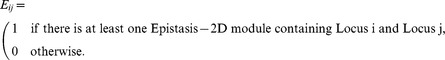



We performed hierarchical clustering on this map using Cluster 3.0.

### Functional enrichment analysis for all gene pairs in the modules

For each gene, we used GO slim to annotate its function. The gene pair in each Epistasis-2D module were annotated with a pair of functions. To investigate whether a particular pair of functions were over-represented among the Epistasis-2D modules, we performed the following hypergeometric test,
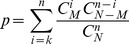
(9)where N is the total number of gene pairs, M is the number of gene pairs with two specific functions, n is the number of gene pairs from Epistasis-2D modules, and k is the number of Epistasis-2D gene pairs having the specific two functions. The p-values were Bonferroni corrected for multiple testing.

Before analyzing the results, we note that many genes involved in these function categories are overlapped. Under the glucose condition, 18 genes annotated as “precursor metabolites/energy” actually consist of carbohydrate metabolic genes (7/18) and cellular respiration genes (11/18). Genes annotated as “cofactor metabolic” are also highly overlapped with these two processes (7/13). In addition, genes annotated as “RNA metabolic process” are mainly involved in ribosome biogenesis (33/80). Under the ethanol condition, most genes annotated as “transcription” and “chromosome organization” are involved in the RNA metabolic process (32/34, 9/19). According to these overlaps, the main differences between the two conditions can be summarized as shown in Figure 4.

### Functional enrichment analysis for chromosome intervals

Since a chromosomal interval encompassing the markers may contain multiple candidate genes, we need to perform enrichment analysis to investigate whether there is statistically significant evidence for the enrichment of certain function. We performed hypergeometric test to investigate whether a particular function was over-represented among the genes located at the chromosomal intervals within one or several modules. The p-value was calculated as,
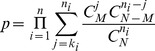
(10)where n is the total number of considered chromosome intervals, N is the total number of annotated genes, M is the number of genes with specific function, 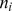 is the number of genes in ith chromosome interval, and 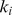 is the number of genes having the specific function in ith chromosome interval. The gene function is defined by GO annotation at level 5 (downloaded from DAVID Knowledgebase [71], [72]). The calculation detail of the examples that discussed in the results section was listed in Table S6.

## Supporting Information

Figure S1 Examples showing the effect of normal quantile transformation. (A) Example 1 with an outlier which overestimates the correlation. (B) Normal quantile transformation (NQT) of Example 1 can reduce the effect of the outlier. (C) Example 2 with an outlier which underestimates the correlation. (D) Normal quantile transformation can reduce the effect of the outlier.
(EPS)

Figure S2 Comparison of correlation coefficients calculated from the original data and transformed data. As shown in the figure, there is a high degree of correlation between gene expression correlation coefficients calculated from normalized and unnormalized data.
(EPS)

Figure S3 Comparison of estimating standard deviation from MLE and using SD = 0.97. (A) For the identified Epistasis-2D modules with large LR statistics, the LR statistics calculated from the two methods are highly correlated. (B) For randomly sampled modules with relative low LR statistics, the LR statistics calculated from the two methods are also highly correlated. (C) The distribution of the estimated standard deviations for the Epistasis-2D modules. (D) The distribution of the estimated standard deviations for random sampled modules.
(EPS)

Figure S4 Comparison of the statistical power of model (3) and model (6) using simulated data. (A) For simulated IMDVED modules with 100 samples, using model (6) resulted in higher power than model (3) for 54% of the modules. (B) For simulated IMIVED modules with 100 samples, using model (6) resulted in lower power than model (3) for 88% of the modules. (C) For simulated IMDVED modules with 500 samples, using model (6) resulted in higher power for 89% of the modules. (D) For simulated IMIVED modules with 500 samples, using model (6) resulted in lower power than model (3) for 94% of the modules.
(EPS)

Figure S5Comparison of the statistical power of model (3) versus model (6) using real data. For modules with 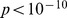 based on the LR tests, using model (6) resulted in lower power than model (3) for 73% of the modules.(EPS)

Figure S6 Comparison of the statistical power of model (3) and model (20) using simulated data. (A) For simulated DMIVED modules with 100 samples, using model (20) led to higher power than model (3) for 58% of the modules. (B) For simulated IMIVED modules with 100 samples, using model (20) resulted in lower power than model (3) for 88% of the modules. (C) For simulated DMIVED modules with 500 samples, using model (20) led to higher power for 92% of the modules. (D) For simulated IMIVED modules with 500 samples, using model (6) resulted in lower power than model (3) for 96% of the modules.
(EPS)

Figure S7Comparison of the statistical power of model (3) and model (20) using real data. For modules with 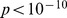 in the LR tests, using model (20) resulted in lower power than model (3) for 96% of the modules.(EPS)

Figure S8Evaluation of PA score and sensitivity estimation. (A) For simulated IMIVED modules, the LR scores and PA scores are highly correlated. (B) For simulated IMDVED modules, the correlation between LR scores and PA scores is lower than that in IMIVED modules. (C) For simulated DMIVED modules, the correlation between LR scores and PA scores is also lower than that in IMIVED modules. (D) For simulated negative controls, the correlation between LR scores and PA scores is much lower than that in IMIVED modules. (E) The correlation between LR scores and PA scores in sampled modules from yeast dataset. (F) For simulated IMIVED modules and different threshold c, the fraction of modules with PA - LR 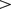 -c is relatively robust to the LR score level. (G) IMIVED modules have higher fraction of modules with PA - LR 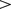 -c for different threshold c. (H) Distribution of the difference between the PA scores and LR scores in yeast data. As shown in the figure, PA-LR = −5.8 (dash line) is the 0.005 quantile.(EPS)

Table S1 Parameter settings.
(PDF)

Table S2Number of unique 2D-traits in significant modules (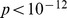) in each permutated dataset.(XLSX)

Table S3 FDRs under different cutoffs.
(XLSX)

Table S4 Epistasis-2D modules.
(XLSX)

Table S5 “RNA metabolic-RNA metabolic” co-regulated modules.
(XLSX)

Table S6 Functional enrichment analysis for Epistasis-2D modules.
(XLSX)

Text S1 Supplementary methods and simulation study.
(PDF)
