# Supplementary material for: Statistical Analysis Reveals Co-Expression Patterns of Many Pairs of Genes in Yeast Are Jointly Regulated by Interacting Loci
Source: PLoS Genet. 2013 Mar 28;9(3):e1003414. doi: 10.1371/journal.pgen.1003414 (PMC3610942; doi:10.1371/journal.pgen.1003414)
Supplement: Table S1 — Parameter settings. (PDF) [file pgen.1003414.s009.pdf]

Setting	I(A = 1) I(B = 1)	I(A = 1) I(B = 0)	I(A = 0) I(B = 1)	I(A = 0) I(B = 0)	Association type
1	β_0	β_0	β_0	β_0	Independant
2	β_1	β_0	β_0	β_0	Epistasis
3	β_0	β_1	β_0	β_0	Epistasis
4	β_0	β_0	β_1	β_0	Epistasis
5	β_0	β_0	β_0	β_1	Epistasis
6	β_1	β_1	β_0	β_0	Single
7	β_1	β_0	β_1	β_0	Single
8	β_1	β_0	β_0	β_1	Epistasis
9	β_1	β_2	β_0	β_0	Epistasis
10	β_1	β_0	β_2	β_0	Epistasis
11	β_1	β_0	β_0	β_2	Epistasis
12	β_0	β_1	β_2	β_0	Epistasis
13	β_0	β_1	β_0	β_2	Epistasis
14	β_0	β_0	β_1	β_2	Epistasis
15	β_0	β_1	β_2	β_3	Epistasis

Table S1. Parameter settings.
