# Supplementary material for: Statistical Analysis Reveals Co-Expression Patterns of Many Pairs of Genes in Yeast Are Jointly Regulated by Interacting Loci
Source: PLoS Genet. 2013 Mar 28;9(3):e1003414. doi: 10.1371/journal.pgen.1003414 (PMC3610942; doi:10.1371/journal.pgen.1003414)
Supplement: Text S1 — Supplementary methods and simulation study. (PDF) [file pgen.1003414.s015.pdf]

Statistical Analysis Reveals Co-Expression Patterns of Many Pairs of Genes in Yeast are Jointly Regulated by Interacting Loci

Lin Wang[1,2], Wei Zheng[2], Hongyu Zhao[2*] & Minghua Deng[1,3,4*]
1 Center for Quantitative Biology, Peking University, Beijing 100871, China.
2 Department of Biostatistics, Yale School of Public Health, New Haven, Connecticut, 06510, USA.
3 LMAM, School of Mathematical Sciences, Peking University, Beijing 100871, China.
4 Center for Statistical Science, Peking University, Beijing 100871, China.
∗ Corresponding authors: hongyu.zhao@yale.edu; dengmh@pku.edu.cn

Introduction

We first review the general workflow of our approach. For a candidate module consisting of two loci and two genes, we first evaluate the likelihood that the module is statistically significant based on a statistic called PA-score (Potential of Association) and filter out modules whose corresponding PA-scores do not pass a given cutoff value. Then we filter out modules where the association can be detected using marginal expression levels without considering co-expression patterns (1D-trait). **We note that for modules where the expressions are affected by markers from the same module in the 1D-Map, the order of applying these two processes does not affect the result since these modules will also be filtered out in our method. The reason that we apply the PA-score filtering first is mostly because the cutoff of PA-score is more stringent than that of 1D-Map. In this case, applying the PA-score filtering first could filter out more modules and leading to a more efficient algorithm. For modules where the expressions are affected by markers outside of the module, they will not be filtered since the correlation between the two genes may be affected by markers within the module. For example, for the "*GPG1-RNT1*" module discussed in the main text, the expression levels of *GPG1* and *RNT1* are affected by a marker *IRA2* in the 1D-Map, but their correlation is also jointly affected by two markers *SSN8* and *GCR1* in the module. We thought these modules may also be meaningful.** After the two filtering steps, we model the co-expression patterns of two traits (2D traits) by a conditional bivariate model. We perform formal statistical tests to identify modules consisting of gene pairs whose co-expression patterns are under interactive control of two loci. In this supplement, we will first introduce the conditional bivariate model and then describe how to calculate p-values and how to distinguish single linkage and epistasis. We also discuss the construction of the PA-score.

Conditional bivariate model

As introduced in the Method section of the main text, the conditional bivariate model is,

$$\begin{pmatrix} X \\ Y \end{pmatrix} |(A,B) \sim \mathbf{N}(\begin{pmatrix} \mu_1 \\ \mu_2 \end{pmatrix}, \Sigma(\beta_{ij}, A, B)) \quad (1)$$

The parameters $\theta = (\beta_{ij}, \sigma_1, \sigma_2)$ in (1) can be estimated using the maximum likelihood estimates (MLE), where the log-likelihood function is,

$$l(\theta) = -\frac{1}{2}\sum_{k=1}^{n}\{log[(1-\rho^2(\beta_{ij}, a_k, b_k))\sigma_1^2\sigma_2^2] + \frac{1}{1-\rho(\beta_{ij}, a_k, b_k)}[\frac{x_k^2}{\sigma_1^2} + \frac{y_k^2}{\sigma_2^2} - \frac{2\rho(\beta_{ij}, a_k, b_k)x_k y_k}{\sigma_1\sigma_2}]\}. \quad (2)$$

Note that we set μ_1 and μ_2 to be 0 for simplification.

Before analyzing the data, we applied the normal quantile transformation to the expression for each gene. The normal quantile transformation is a means to "normalize" the sample observations so that our procedure is robust to the effects of extreme observations and/or highly skewed distributions. Figures S1A-D show the effect of normal quantiale transformation on two examples with outliers from the yeast data, where the correlations estimated from the transformed data are more reasonable. For most situations, we expect the normal quantile transformation should have little effect on the estimation of the correlation compared to using original data. To examine this, we calculated the correlation coefficients between all pairs of genes in the yeast data using both original and normal quantile transformed expression. We can see from Figure S2 that the correlation coefficients estimated from the two groups of expression data are quite consistent (Cor = 0.99). Hence, our methods will be relatively robust to small to mild departures from the normal assumptions. We should note that the normal quantile transformation could not guarantee the bi-variate normal distribution. For example, when the gene expression levels are under the genetic control, the joint distribution of the gene expressions follows the conditional bi-variate normal but their unconditional joint distribution will not be bi-variate normal, which is the case for Epistasis-2D modules identified in this paper. When the genetic effects are not large, we expect the bi-variate assumption may be a good approximation. For example, we randomly sampled 10^6 pairs of genes from the yeast data which mostly could be considered as genotype-independent gene pairs, and tested their joint distribution using the normal quantile transformed expressions data. We used the (R) function mvShapiro.Test within R package mvShapiroTest to perform the Shapiro-Wilk test to evaluate multivariate normality ([1]). The joint normal distribution assumption cannot be rejected at the 0.05 statistical significance level for 99.95% of the gene pairs.

Because we quantile normalize the data, the overall standard deviation of each expression trait is 0.97. Therefore, one possible strategy is to fix the standard deviation at 0.97. We have chosen to allow the variance to be estimated instead and note that this will have minimal impact on the results as the likelihood ratio test has the same degrees of freedom when either the standard deviation is fixed or estimated. More specifically, we took two groups of modules to compare the performance of these two alternative strategies, one by randomly sampling 10^5 modules and another by considering Epistasis-2D modules with p-values lower than 10^{-10} in the LR tests. These two groups of modules represent modules with different significant levels in LR tests. As shown Figure S3A-B, there was a good consistency between the LR statistics using estimated variance and fixed variance for both groups. Further more, the statistic based on estimated variance was in general larger than that with fixed variance. The estimated standard deviations were also close to 0.97 (0.94 $\sim$ 1) in both groups (Figure S3C-D). We used the **R** function **nlm** for the likelihood estimation in our analysis.

Even when L_1 and L_2 interact to regulate G_1 and G_2, the β_{ij} are not necessarily different. In this case, using the model above may reduce the statistical power for detecting such associations because more parameters are used in the model. To select the best model for testing, we consider all 15 possible parameter settings for epistatic interactions (Table S1). We can see that setting 1 corresponds to no linkage between L_1, L_2 and G_1, G_2 (independant), settings 6 and 7 imply that the co-expression pattern of G_1 and G_2 only depend on L_1 or L_2 (single linkage) and the other 12 settings imply epistasis. We compare the 12 alternative models with the independant model (setting 1) to infer the presence of epistatic effects between L_1 and L_2 on the co-expression patterns between G_1 and G_2. We used the following likelihood ratio (LR) test:

$$-2l(\hat{\theta}_0) + 2l(\hat{\theta}_i) \sim \chi^2(df_i - df_0), i = 1, ..., 12 \quad (3)$$

for comparisons where $\hat{\theta}_0$ and $\hat{\theta}_i$ represent the parameters estimated using MLE, df_0 and df_i correspond to the degrees of freedom under the null model and the alternative model. Among 12 comparisons, we selected the most significant model to describe each module. To identify significant modules, we selected a threshold C, and call all the modules "significant" if their p-values were less than the cutoff value. For modules passing the cutoff, we further compared the selected model with the two single linkage models (setting 6,7) to see whether L_1 and L_2 have epistatic effects. If the degrees of freedom of the selected model were equal to 2 (as the single linkage models), we compared the single linkage models with the independant model. If the smaller p-value of the two single linkage models also passed the cutoff C, this module would no longer be considered as candidates for having epistatic effects. If the degrees of freedom in the selected model were 3 or 4, we used the LR test to compare this model with the two single linkage models. If the larger p-value of the two tests was less than 10^{-4}, the module would be retained. Considering that there are $\sim 3 \times 10^3$ loci in the yeast dataset, this cut-off leads to an average 0.3 false positive results.

Filtering process

Construction of PA score

Since maximum likelihood estimates (MLEs) need to solve a numerical optimization problem, applying the tests above to all possible modules is computationally expensive. One possible solution is to construct a simplified statistic to filter modules with low possibility of being an Epistasis-2D module. Since a formal assessment is based on the LR test (3), if we could find one statistic $S(A, B, X, Y)$, such that

$$S(A, B, X, Y) > -2l(\hat{\theta}_0) + 2l(\hat{\theta}_i) \tag{4}$$

for any i, then if $S(A, B, X, Y) < \chi^2(1-p_0, 1)$, the p-value for this module is larger than p_0. To find such statistic we first consider the full model where the variances σ_1, σ_2 also depend on A, B, that is,

$$\begin{cases} \sigma_1(\beta^1_{ij}, A, B) = \sum_{i\in T, j\in T} \beta^1_{ij} I(A = i)I(B = j) \\ \sigma_2(\beta^2_{ij}, A, B) = \sum_{i\in T, j\in T} \beta^2_{ij} I(A = i)I(B = j) \end{cases} \tag{5}$$

It is obvious that the likelihood of this model is larger than any of the 12 models, hence,

$$S(A, B, X, Y) = -2l(\hat{\theta}_0) + 2l(\hat{\theta}_{full}) \tag{6}$$

satisfying (4).

Actually, the MLE of $\hat{\theta}_0$ under the null model can be directly calculated as,

$$\hat{\sigma}_1 = \frac{1}{n}\sum_{i=1}^{n} x_i^2$$
$$\hat{\sigma}_2 = \frac{1}{n}\sum_{i=1}^{n} y_i^2$$
$$\hat{\rho} = \frac{\frac{1}{n}\sum_{i=1}^{n} x_i y_i}{\hat{\sigma}_1 \hat{\sigma}_2} \tag{7}$$

then we have

$$\begin{aligned}
-2l(\hat{\theta}_0) &= \sum_{k=1}^{n}\{log[(1-\hat{\rho}^2)\hat{\sigma}_1^2\hat{\sigma}_2^2] + \frac{1}{1-\hat{\rho}^2}[\frac{x_k^2}{\hat{\sigma}_1^2} + \frac{y_k^2}{\hat{\sigma}_2^2} - \frac{2\hat{\rho}x_ky_k}{\hat{\sigma}_1\hat{\sigma}_2}]\} \\
&= \sum_{k=1}^{n} log[(1-\hat{\rho}^2)\hat{\sigma}_1^2\hat{\sigma}_2^2] + \frac{1}{1-\hat{\rho}^2}[\frac{\sum_{k=1}^{n} x_k^2}{\hat{\sigma}_1^2} + \frac{\sum_{k=1}^{n} y_k^2}{\hat{\sigma}_2^2} - \frac{2\hat{\rho}\sum_{k=1}^{n} x_ky_k}{\hat{\sigma}_1\hat{\sigma}_2}]\} \\
&= nlog[(1-\hat{\rho}^2)\hat{\sigma}_1^2\hat{\sigma}_2^2] + \frac{1}{1-\hat{\rho}^2}[n+n-2n\hat{\rho}^2] \\
&= nlog[(1-\hat{\rho}^2)\hat{\sigma}_1^2\hat{\sigma}_2^2] + 2n \qquad (8)
\end{aligned}$$

For the full model, we can partition the likelihood function as,

$$\begin{aligned}
2l(\hat{\theta}_{full}) &= -\sum_{k=1}^{n}\{log[(1-\rho^2(\beta_{ij},a_k,b_k))\sigma_1^2(\beta_{ij}^1,a_k,b_k)\sigma_2^2(\beta_{ij}^2,a_k,b_k)]+ \\
&\quad \frac{1}{1-\rho^2(\beta_{ij},a_k,b_k)}[\frac{x_k^2}{\sigma_1^2(\beta_{ij}^1,a_k,b_k)} + \frac{y_k^2}{\sigma_2^2(\beta_{ij}^2,a_k,b_k)} - \frac{2\rho(\beta_{ij},a_k,b_k)x_ky_k}{\sigma_1(\beta_{ij}^1,a_k,b_k)\sigma_2(\beta_{ij}^2,a_k,b_k)}]\}. \\
&= -\sum_{i,j\in T}\sum_{k\in D_{ij}}\{log[(1-\rho_{ij}^2)\sigma_{1,ij}^2\sigma_{2,ij}^2] + \frac{1}{1-\rho_{ij}^2}[\frac{x_k^2}{\sigma_{1,ij}^2} + \frac{y_k^2}{\sigma_{2,ij}^2} - \frac{2\rho_{ij}x_ky_k}{\sigma_{1,ij}\sigma_{2,ij}}]\}, \qquad (9)
\end{aligned}$$

where D_{ij} denotes the individuals set with genotype $A = i$ and $B = j$, and

$$\begin{aligned}
\sigma_{1,ij} &= \sigma_1 I(A=i)I(B=j) = \beta_{ij}^1 \\
\sigma_{2,ij} &= \sigma_2 I(A=i)I(B=j) = \beta_{ij}^2 \\
\rho_{ij} &= \rho I(A=i)I(B=j) = \beta_{ij}
\end{aligned}$$

This suggests that we can estimate the parameters separately based on their genotype. We note that for given $i, j \in T$ the likelihood function is equal to the null model likelihood function. Hence, similar to (7), we have,

$$\begin{aligned}
\hat{\sigma}_{1,ij} &= \frac{1}{n_{ij}}\sum_{k\in D_{ij}}(x_k-\bar{x}_{ij})^2 \\
\hat{\sigma}_{2,ij} &= \frac{1}{n_{ij}}\sum_{k\in D_{ij}}(y_k-\bar{y}_{ij})^2 \\
\hat{\rho_{ij}} &= \frac{\frac{1}{n_{ij}}\sum_{k\in D_{ij}}(x_k-\bar{x}_{ij})(y_k-\bar{y}_{ij})}{\hat{\sigma}_{1,ij}\hat{\sigma}_{2,ij}} \qquad (10)
\end{aligned}$$

where n_{ij} is the number of individuals with genotypes $A = i, B = j$, $\bar{x}_{ij}, \bar{y}_{ij}$ is the mean values of the expression levels. Although we performed normal score transformation, the means of $\bar{x}_{ij}, \bar{y}_{ij}$ may not be 0. Then similar to (8), we have,

$$2l(\hat{\theta}_{full}) = -\sum_{i,j\in T} n_{ij}log[(1-\hat{\rho}_{ij}^2)\hat{\sigma}_{1,ij}^2\hat{\sigma}_{2,ij}^2] - 2n \qquad (11)$$

Combining (8,11) we get,

$$\begin{aligned}
S(A,B,X,Y) &= -2l(\hat{\theta}_0) + 2l(\hat{\theta}_{full}) \\
&= nlog[(1-\hat{\rho}^2)\hat{\sigma}_1^2\hat{\sigma}_2^2] + 2n - \sum_{i,j\in T} n_{ij}log[(1-\hat{\rho}_{ij}^2)\hat{\sigma}_{1,ij}^2\hat{\sigma}_{2,ij}^2] - 2n \\
&= nlog[(1-\hat{\rho}^2)\hat{\sigma}_1^2\hat{\sigma}_2^2] - \sum_{i,j\in T} n_{ij}log[(1-\hat{\rho}_{ij}^2)\hat{\sigma}_{1,ij}^2\hat{\sigma}_{2,ij}^2] \\
&= nlog(1-\hat{\rho}^2) - \sum_{i,j\in T} n_{ij}log(1-\hat{\rho}_{ij}^2) \qquad (12) \\
&+ nlog\hat{\sigma}_1^2 - \sum_{i,j\in T} n_{ij}log\hat{\sigma}_{1,ij}^2 \qquad (13) \\
&+ nlog\hat{\sigma}_2^2 - \sum_{i,j\in T} n_{ij}log\hat{\sigma}_{2,ij}^2 \qquad (14)
\end{aligned}$$

Now we decompose $S(A,B,X,Y)$ into three parts (12), (13) and (14), which reflect the variance of ρ, σ_1, σ_2 for individuals having different genotypes. As discussed above, we assume that σ_1, σ_2 are the same for different genotypes. Under this assumption, the expectation of (13,14) is 0. Therefore, we have arrived at the statistic (12) and name it as "PA-score" (Potential of Association),

$$PA = nlog(1-\hat{\rho}^2) - \sum_{i,j\in T} n_{ij}log(1-\hat{\rho}_{ij}^2). \qquad (15)$$

Therefore, the expectation of PA is the upper bound of -2l($\hat{\theta}_0$)+2l($\hat{\theta}_i$). **If the upper bound of LR statistic for one module could not pass the expected cutoff of the LR tests, then this module will not be significant in the LR test. Although it is the expectation of PA not PA that is the upper bound, we could give a lower cutoff of PA score to ensure only few modules that could be significant in the LR tests to be filtered out by PA score.** This statistic may be better than $S(A,B,X,Y)$ because it also helps to filter out modules with large variance of σ_1 or σ_2 but low variance of ρ.

Sensitivity

For the yeast dataset considered in the manuscript, there are a total of 3×10^{12} candidate modules. With a statistical significance threshold values of 10^{-12}, we expect an average of 3 false positive results. Across the 12 alternative models considered, the numbers of degrees of freedom for the 12 models vary from 4 to 6 whereas the null model has 3 degrees of freedom, the threshold for the likelihood ratio statistic should at least be $\chi^2(1-10^{-12}, 4-3) = 50.8$. **To study the number of modules which are significant in LR tests but may be filtered out by PA score, we define the sensitivity of PA filter as the fraction of significant modules in LR tests that will not be filtered out by PA score. We estimate the sensitivity level from the empirical data through the following steps.**

- Selecting 10^5 modules from the yeast dataset;
- Calculating PA-score for each module;
- Calculating LR statistic from model (3) for setting 15 in Table S1.

Then we estimated the sensitivity of our filtering process for a given level c by

$$\texttt{Sensitivity}_c = \frac{\#\{\texttt{All modules with PA-LR>-c}\}}{\#\{\texttt{All modules}\}} \qquad (16)$$

The rationale of this estimation is further discussed in the following simulation study section. For a threshold value of c=5.8, we estimated that the sensitivity to be larger than 0.995 from the distribution of PA-LR (Figure S8H). Hence we use a cutoff value of 50.8-5.8=45 for PA with an estimated sensitivity level larger than 0.995. **Because our procedure detected ~ 225 modules, we estimate that there may be $225 \times \frac{0.005}{0.995} \approx 1$ module missed by PA score in each condition.**

Computational efficiency

To estimate how much faster the filtering process can be, we randomly selected 10000 modules and test the computational time for calculating the PA and fiting the full model. The result showed a reduction of 16 fold with the filtering step.

1D-map filtering

Since the PA-score does not filter associations that can be detected with 1D traits, we applied the Wilcoxon test to the remaining modules and retained only those with non-significant ($p > 0.001$) marginal signals to perform the LR tests.

Simulation studies

We conducted simulations to investigate the impacts of departures from our modeling assumptions, i.e. genotype independent means and variances, on the power to detect Epi-2D modules. We simulated 100 samples for each module (A, B, X, Y), and generated 10,000 groups of modules where each group contained one genotype-Dependent Mean values but genotype-Independent Variances Epi-2D module (DMIVED), one genotype-Independent Mean values but genotype-Dependent Variances Epi-2D (IMDVED) module and one genotype-Independent Mean values and genotype-Independent Variances Epi-2D (IMIVED) module. The three modules in each group shared the same correlation coefficients for X and Y in the conditional bi-variate distributions, and only differed in their mean values and/or variances as detailed below. We also simulated 10,000 negative controls where the correlation between X and Y is the same for all samples. We considered the following set-up in our simulations:

- **For all modules, we let**

$$A = \begin{cases} 0 & \texttt{for sample 1-50} \\ 1 & \texttt{for sample 51-100} \end{cases} \quad B = \begin{cases} 0 & \texttt{for sample 1-25 and 51-75} \\ 1 & \texttt{for sample 26-50 and 76-100} \end{cases} \tag{17}$$

  This way there is an equal number of individuals, i.e. 25, for the four possible genotype groups: 1-25, 26-50, 51-75, and 76-100.

- **For each group of modules, we simulated the four correlation coefficients between X and Y in each of the four joint genotype groups from a uniform [-0.9,0.9] distribution.**

- **For the IMDVED and IMIVED modules in each group, the mean value was set to be 0 for all four genotypes groups. For the DMIVED modules, the mean values for X and Y in each group were simulated from a uniform [-0.5,0.5] distribution.**

- **For the DMIVED and IMIVED modules in each group, the standard deviation was set to be 1 for all four genotypes groups. For the IMDVED modules, the standard deviations for X and Y in each group were simulated from a uniform [0.5,1.5] distribution.**

- **For negative controls, there is only one correlation coefficient in each module. We also simulated the correlation coefficients from a uniform [-0.9,0.9] distribution.**

- **The gene expression levels for the 100 individuals in each module were simulated from their conditional bi-variate distributions defined by their joint genotypes.**
- **We scaled the expressions for each module using normal quantile transformation as described in the method section of the main text to ensure the statistics calculated below are comparable between modules.**

Genotype-dependant variance assumption

To investigate the potential power loss under the genotype-independent variance assumption made in this paper, we compared the statistical power for detecting IMIVED and IMDVED modules using data simulated as described above. We compared the performance between the full model (6) where the variances were allowed to be genotype dependent versus our proposed genotype-independent variance model (3). As clearly shown in Figures S4A-B, although using full model (6) led to some increased power to detect IMDVED modules, it may substantially reduce the power to detect IMIVED. This is because using the full model (6) rather than our model (3) makes use of more parameters (9 versus 3) and leads to power loss even with the presence of mild departure from the genotype-independent variance assumption. To examine the effect of sample size, we also simulated another dataset where the sample size was increased from 100 to 500 while the other settings were the same. In this dataset, there was an increased power gain using the full model (6) to detect IMDVED modules (Figure S4C), although the power to detect IMIVED was still much lower (Figure S4D). This indicates that when more samples are available and there is some indication of genotype-dependant variances, our model may be extended to detect more complex modules like IMDVED modules.

We have also performed the comparison using Epistasis-2D modules with p-value lower than 10^{-10} in LR tests (3) in yeast data, which could be mostly considered as IMIVED modules in real data. As shown in Figure S5, using full model (6) indeed substantially reduced the power as in simulated data.

Genotype-dependant mean value assumption

The main reason we assumed genotype-independent mean values is that this kind of associations can be easily identified by 1D-trait mapping and is not our interest of this paper. In addition, assuming genotype-dependent mean values will also introduce extra parameters into the model which may reduce the statistical power for identifying IMIVED modules. Similar to genotype-independent variance assumption analysis, we compared the statistical power for detecting DMIVED and IMIVED modules using simulated data to investigate the potential power loss under the genotype-independent mean value assumption. We considered a model which assumed that the mean values in model (1) are genotype dependent to detect DMIVED modules, that is:

$$\begin{cases} \mu_1(\alpha^1_{ij}, A, B) = \sum_{i\in T, j\in T} \alpha^1_{ij} I(A=i)I(B=j) \\ \mu_2(\alpha^2_{ij}, A, B) = \sum_{i\in T, j\in T} \alpha^2_{ij} I(A=i)I(B=j) \end{cases} \tag{18}$$

The parameters $\theta_{mean} = (\alpha^1_{ij}, \alpha^2_{ij}, \beta_{ij}, \sigma_1, \sigma_2)$ in this model can be estimated using MLE where the log-likelihood function is,

$$\begin{aligned} l(\theta_{mean}) = & -\frac{1}{2}\sum_{k=1}^{n}\{log[(1-\rho^2(\beta_{ij}, a_k, b_k))\sigma_1^2\sigma_2^2] + \frac{1}{1-\rho(\beta_{ij}, a_k, b_k)}[\frac{(x_k-\mu_1(\alpha^1_{ij}, a_k, b_k))^2}{\sigma_1^2} \\ & + \frac{(y_k-\mu_2(\alpha^2_{ij}, a_k, b_k))^2}{\sigma_2^2} - \frac{2\rho(\beta_{ij}, a_k, b_k)(x_k-\mu_1(\alpha^1_{ij}, a_k, b_k))(y_k-\mu_2(\alpha^2_{ij}, a_k, b_k)))}{\sigma_1\sigma_2}]\}. \end{aligned} \tag{19}$$

Then we can use the following LR test:

$$-2l(\hat{\theta}_0) + 2l(\hat{\theta}_{mean}) \tag{20}$$

for comparison.
As shown in Figures S6A-B, using the above model (20) led to increased power to detect DMIVED modules, but it also substantially reduces the power to identify IMIVED. We also simulated another dataset with 500 samples while the other settings were the same. As expected, there was a significant increased power gain using model (20) to detect DMIVED modules (Figure S6C), although its power to detect IMIVED was still much lower (Figure S6D).

Similar to genotype dependent variance analysis, we have also performed the comparison using Epistasis-2D modules with p-value lower than 10^{-10} in LR tests (3) in yeast data. We also found that using model (20) reduced the power as illustrated in the simulated data (Figure S7).

Evaluating PA score and sensitivity estimation

To evaluate the efficiency of using the PA score as a filtering criterion, we investigated the relationship between the PA and LR scores. As expected, for the IMIVED modules, the PA scores and LR scores were highly correlated (cor = 0.99, Figure S8A). Hence, we could give a lower cutoff of the PA score to minimize the number of modules that could be significant in the LR tests to be filtered out by the PA score. More formally, we could choose c so that $LR - PA < c$ for most modules. Then for a given cutoff C, most modules with $PA < C - c$ will have $LR < C$. For IMDVED modules, DMIVED modules and negative controls, the PA scores and LR scores were also correlated, but the correlation was lower (cor = 0.97, cor = 0.95, cor = 0.74, Figures S8B-D). We also sampled 10,000 modules from the yeast dataset and calculated their PA and LR scores. Their LR-PA plot resembled more of that of the negative controls (Figure S8E), because the correlations between X and Y in most modules were expected to be independent of the genotypes. In fact, we could consider the modules from the real data as a mixture of different types of modules. If we want to estimate the sensitivity (how many modules that could be significant in LR test would pass the PA filter) from the empirical data, we need to know its properties across different types of modules. First, for a given type of modules, the difference between LR and PA should be independent of the value of the LR score. That is,

$$\frac{\#\{\texttt{IMIVED modules with LR>C, PA-LR>-c}\}}{\#\{\texttt{IMIVED modules with LR>C}\}} \approx \frac{\#\{\texttt{IMIVED modules with PA-LR>-c}\}}{\#\{\texttt{All IMIVED modules}\}} \tag{21}$$

This is because their difference mainly depends on the variance terms (13,14) which is not related to the LR level. This conclusion is supported by the simulated data (Figure S8F). Hence we could use all the data without regarding their LR level to estimate the sensitivity. Second, since the correlations between PA and LR are higher for IMIVED modules, the difference between PA and LR should be smaller. Specially, we should have

$$\frac{\#\{\texttt{IMIVED modules with PA-LR>-c}\}}{\#\{\texttt{All IMIVED modules}\}} \geq \frac{\#\{\texttt{All modules with PA-LR>-c}\}}{\#\{\texttt{All modules}\}} \tag{22}$$

for a positive number c. This is also supported by the simulated data (Figure S8G). Consideration of the above observations led to the following conservative method to estimate sensitivity from the empirical data for IMIVED modules: assuming the cutoff for LR score

and PA score is C and C-c, respectively, then:

$$
\begin{aligned}
\texttt{Sensitivity}_c &= \frac{\#\{\texttt{IMIVED modules with LR>C, PA>C-c}\}}{\#\{\texttt{IMIVED modules with LR>C}\}} \\
&\geq \frac{\#\{\texttt{IMIVED modules with LR>C, PA-LR>-c}\}}{\#\{\texttt{IMIVED modules with LR>C}\}} \\
(21) &\approx \frac{\#\{\texttt{IMIVED modules with PA-LR>-c}\}}{\#\{\texttt{All IMIVED modules}\}} \\
(22) &\geq \frac{\#\{\texttt{All modules with PA-LR>-c}\}}{\#\{\texttt{All modules}\}}
\end{aligned}
\tag{23}
$$

which was used in our estimation above.

False discovery rate estimation

Finally, we estimated the FDR through the permutation test described in the method section of the main text. The results for the ten permutations in each condition are listed in Table S2. The FDR is defined as

$$
\texttt{FDR}_c = \frac{\#\texttt{of unique traits in Epi-2D modules with p-value<C in permutated dataset}}{\#\texttt{of unique traits in Epi-2D modules with p-value<C in real dataset}} \tag{24}
$$

Table S3 lists the FDRs under different cutoffs for each condition. We adopted 10^{-12} as the cutoff value so that the FDR was < 0.2 for both conditions, and a total of 225 and 224 significant 2D-traits were identified.

References

1. Villaseñor Alva JA, González-Estrada E (2009) A bootstrap goodness of fit test for the generalized pareto distribution. Comput Stat Data Anal 53: 3835–3841.
